# Opinion of speech-language pathologist on the use of photobiomodulation in the vocal clinic

**DOI:** 10.1590/2317-1782/20232022060en

**Published:** 2023-09-18

**Authors:** Emerson Soares Pontes, Thays Garcia Vaiano, Roberto Sávio de Assunção Bastos, Leonardo Wanderley Lopes

**Affiliations:** 1 Universidade Federal da Paraíba - UFPB - João Pessoa (PB), Brasil.; 2 Centro de Estudos da Voz - CEV - São Paulo (SP), Brasil.; 3 Hospital Pronto Socorro Municipal Mário Pinotto - Belém (PA), Brasil.

**Keywords:** Voice, Low-level Light Therapy, Voice Disorders, Voice Training, Laryngeal Diseases

## Abstract

**Purpose:**

to investigate the opinion of Brazilian speech-language pathologists on the training, performance, and parameters used for the application of photobiomodulation (PBM) in the vocal clinic.

**Methods:**

observational, cross-sectional, and quantitative study, carried out through a web survey hosted on the Google Forms digital platform, composed of questions related to training, professional performance, and knowledge about PBM in the voice area. Twenty-nine speech-language pathologists of both sexes participated. Data were analyzed using descriptive statistics.

**Results:**

all participants knew the theoretical foundations of PBM, and among them, 28 (96.6%) knew its use specifically in the voice area; twenty-five respondents (86.2%) had a device to perform the irradiation, and all of them used it routinely in their clinical practice in voice. The majority (96.6%, 28) participated in a PBM training course, including specific approaches to the voice area. Participants stated that PBM is a resource that can be used in the area of ​​voice to improve performance in sung (86.2%, 25) and spoken (82.8%, 24), in addition to its application in cases of inflammatory processes in the vocal folds (79.3%, 23). As for dosimetry parameters, the most used wavelength was 808 - 830nm (37.9%, 11) and 660/808nm simultaneously (37.9%, 11), with a dose of 3-5 J per point for the patients with inflammatory processes in the vocal folds (51.7%, 15) and 6-9 J (44.8%, 13) per point for patients whose objective was improvement/conditioning.

**Conclusion:**

the study participants demonstrated knowledge and training in PBM and its applicability to the voice area.

## INTRODUCTION

Vocal therapy generally aims to reduce the patient’s symptoms and limitations and improve vocal functioning for everyday voice use situations^(1-4)^. It involves three main components^(3,4)^: a treatment target (the function of the patient aimed to be changed with the ingredient); an ingredient (including clinician’s procedures, using devices, modeling, using words and commands, manipulating the patient during vocal rehabilitation treatment, and meta-therapy elements, all aimed at changing the predefined target); and the action mechanism (understanding of how the ingredient will change the target).

Vocal therapy ingredients can be classified as volitive and non-volitive^(3,4)^. The action mechanism of volitive ones necessarily includes learning new behaviors related to voice use. On the other hand, non-volitive ingredients do not require patients to have any specific action (e.g., doing an exercise) or learn any new vocal behavior (e.g., change some adjustment in the respiratory, phonatory, or resonance/articulatory subsystems).

In vocal therapy, ingredients may include the use of technological devices as a complementary strategy to change predefined targets and reach the expected therapy results^(3,4)^. Thus, photobiomodulation (PBM), considering the taxonomy proposed by Van Stan et al.^(4)^, can be considered a non-volitive device (as it does not require any specific action of the patient) meant to optimize treatment results^(5)^. PBM refers to the capacity of light to induce biological processes in cells, including anti-inflammatory and analgesic effects, decreased edema, tissue recovery, and improved muscle performance^(6,7)^.

PBM has been used in the field of voice for its anti-inflammatory, analgesic, and cell activity-modulating properties. Hypothetically, these properties can help decrease inflammatory processes commonly present in laryngeal lesions and improve muscle performance. However, such effects are so far only hypothetical, by analogy to its effects on other body tissues unrelated to the larynx^(8-10)^. The body of evidence is not enough yet to support recommending PBM to dysphonic patients or vocally healthy occupational voice users who wish to improve their vocal performance. There has been an effort in the last years, though incipient, to research PBM use on the voice^(11,12)^.

Nevertheless, PBM has proved to effectively treat various other health conditions in dentistry, dermatology, physical therapy, otorhinolaryngology, and speech-language-hearing (SLH) therapy^(11,13)^. In 2021, the Federal SLH Council (CFFa) regulated PBM use for SLH therapists to use as a therapy resource. Its states that they can use PBM therapy as a therapy resource associated with conventional SLH clinical procedures. Moreover, the treatment can be used directly and/or indirectly, adapted or transdermic for systemic intervention^(13)^.

Therefore, considering the possible potential of PBM to optimize SLH intervention results in dysphonic patients and vocally healthy individuals, the scarcity of studies supporting external evidence of PBM use in the voice, and the need for understanding the current use of this type of device in vocal clinical practice, this research aimed to investigate the opinion of Brazilian SLH therapists on the training, procedures, and parameters used to apply PBM in vocal clinical practice. It is expected that the study results will help develop clinical references for PBM use in vocal clinical practice and provide insight for future research.

## METHODS

### Study design

This quantitative, cross-sectional, observational study was approved by the Human Research Ethics Committee of the originating institution’s Department of Health Sciences, under evaluation report no. 3.998.709. All study volunteers read and signed an informed consent form, agreeing to participate in the research.

### Participants

Participants were recruited by displaying posters with information and sending a link to access the research via social media of the laboratory where this investigation was carried out. These media have followers in different parts of the country, specifically interested in the area of voice, which helps reach the target public of the research.

The eligibility criteria were as follows: being a professional with a degree in SLH Sciences, working in Brazil in the area of voice. Thus, the convenience sample comprised 29 SLH therapists of both sexes.

### Procedures

The research was conducted via a web survey hosted in Google Forms. Three SLH therapists experienced in applying PBM to the voice were initially interviewed to discuss the main points to be approached to reach the research objectives. These SLH therapists met the following eligibility criteria: being an SLH therapist specialized in voice, with more than 10 years of experience in the area of voice; having participated in theoretical-practical PBM use training, including its applications in the area of voice; having at least 2 years of experience applying PBM to rehabilitate dysphonic patients or train occupational voice users; being specialized in voice. The three selected professionals worked predominantly in clinics and did not teach at any public or private university. A questionnaire was developed based on the interview content, as shown in Chart 1.

**Chart 1:** Questions about photobiomodulation and their respective possibilities of answers

QUESTION	PARTICIPANTS' POSSIBILITY OF RESPONSE
1. - Do you know the precepts of laser therapy/photobiomodulation?	Yes/No
2. - Are you familiar with the use of laser therapy/photobiomodulation in speech-language-hearing therapy, specifically in the area of voice?	Yes/No
3. - Do you have access to low-level laser equipment?	No/Yes, private; Yes, rented/borrowed; Yes, university’s equipment; Other
4. - Do you usually use laser in your clinical practice in the area of voice?	Yes/No
5. - Did you learn about laser therapy/photobiomodulation in your undergraduate classes?	Yes/No
6. - Did you learn about laser therapy/photobiomodulation in your postgraduate classes?	Yes/No
7. - Did you have any training to use laser therapy/photobiomodulation in speech-language-hearing therapy or health in general?	Yes/No
8. - Did you have any training to use laser therapy/photobiomodulation in speech-language-hearing therapy clinically aimed at the area of voice?	Yes/No
9. - Did you have access to the bibliography on laser therapy/photobiomodulation in speech-language-hearing therapy or health in general?	Yes/No
10. - Did you have access to the bibliography on laser therapy/photobiomodulation in the area of voice?	Yes/No
11. - Did you have access to CFFa recommendations on laser therapy/photobiomodulation use by speech-language-hearing therapists?	Yes/No
12. - What are the main cases in which you use laser therapy/photobiomodulation in voice?	Behavioral dysphonia without lesions / Behavioral dysphonia with lesions / Organic neurological dysphonia / Dysphonia due to sequelae of head and neck cancer / Vocal improvement/training (speech) / Vocal improvement/training (singing).
13. - Do you agree that speech-language-hearing therapists specialized in voice can use laser therapy/photobiomodulation?	Yes/No
14. - Do you agree that laser therapy/photobiomodulation is a therapeutic resource indicated for the voice?	Yes/No
15. - Do you use laser therapy/photobiomodulation in individuals with vocal fold inflammatory processes (having edemas, nodules, and/or polyps)?	Yes/No
16. - Do you use laser therapy/photobiomodulation to improve/train vocal performance in speech?	Yes/No
17. - Do you use laser therapy/photobiomodulation to improve/train vocal performance in singing?	Yes/No
18. - At what moment of the vocal therapy do you use laser therapy/photobiomodulation?	Before vocal exercises / During vocal exercises / After vocal exercises / Before and during vocal exercises / During and after vocal exercises / Before, during, and after vocal exercises
19. - What wavelength do you use the most in the laryngeal region?	650-660 nm / 808-830 nm / 904-907 nm / 660 and 808 nm (simultaneously)
20. - What irradiation method do you use the most in the laryngeal region?	Point contact / Point non-contact / Sweeping non-contact
21. - Where do you most often apply laser/LED in the laryngeal region?	Lamina of the thyroid cartilage unilaterally / Lamina of the thyroid cartilage bilaterally / Other
22. - How many points per laser/LED application do you usually use for the voice?	1 / 2 / 3 / 4 / Above 5
23. - What dose of Joules (J) do you use in patients with vocal fold inflammatory processes?	1-3 J / 3-5 J / 6-9 J
24. - What dose of Joules (J) do you use in patients to improve/train their voices?	1-3 J / 3-5 J / 6-9 J
25. - Do you use systemic laser therapy - modified/transdermic ILIB in the area of voice for rehabilitation and/or vocal improvement/training?	Yes/No

Then the link to access the questionnaire was made available on the social media of the laboratory where this research was carried out, as previously described. Data were collected between July and August 2020. The questionnaire had items on the SLH therapists’ sociodemographic profile, training, professional practice, and knowledge of PBM principles and use in the area of voice. Before answering the questions, participants had access to a text explaining the research, then read the informed consent form, and if they agreed with it, proceeded with the answers.

Data were organized and categorized into Microsoft Excel spreadsheets and then analyzed with descriptive statistics.

## RESULTS

The web survey had 29 participating SLH therapists, whose information is available in Table 1.

**Table 1:** Sociodemographic and occupational data of participating speech-language-hearing therapists

Variable	N	%
SEX		
Females	22	75.9
Males	7	24.1
AGE RANGE		
20 - 30 years	9	31.0
31 - 40 years	8	27.6
41 - 50 years	8	27.6
51 - 60 years	4	13.8
SCHOOLING		
Undergraduate	3	10.3
Specialization	16	55.2
Master’s	6	20.7
Doctoral	3	10.3
Not reported	1	3.4
TEACHING SLH COURSES		
Not teaching	19	65.5
teaching undergraduate classes	1	3.4
Teaching postgraduate classes	6	20.7
Both	1	3.4
Not reported	2	6.9
TEACHING POSTGRADUATE CLASSES		
Not teaching	19	65.5
Specialization	5	17.2
Master’s	0	0.0
Both	1	3.4
Not reported	4	13.8
Total	29	100.0

**Caption:** SLH = Speech-language-hearing

All research participants knew the PBM theoretical framework; 28 (96.6%) of them were familiar with its use specifically in the area of voice; 25 (86.2%) had their own device, and all of them normally used it in their voice clinical practice (Table 2).

**Table 2:** Questionnaire data on the knowledge about using photobiomodulation in the area of voice

**Variable**		**N**	**%**	
**Do you know the precepts of photobiomodulation?**				
**Yes**		29	100.0	
**No**		0	0.0	
**Are you familiar with the use of photobiomodulation specifically in the area of voice?**				
**Yes**		28	96.6	
**No**		1	3.4	
**Do you have access to laser or LED equipment?**				
**I do not have access**		3	10.3	
**Yes, I have private equipment.**		25	86.2	
**Yes, I rent/borrow it.**		0	0.0	
**Yes, there is equipment at the university/laboratory**		1	3.4	
**Do you usually use lasers in your clinical practice in the area of voice?**				
**Yes**		25	86.2	
**No**		4	13.8	
**Did you learn about photobiomodulation in your undergraduate classes?**				
**Yes**		3	10.3	
**No**		26	89.7	
**Did you learn about photobiomodulation in your postgraduate classes?**				
**Yes**		7	24.1	
**No**		22	75.9	
**Did you have any training to use laser therapy?**				
**Yes**		28	96.6	
**No**		1	3.4	
**Did you have any training to use photobiomodulation in speech-language-hearing therapy clinically aimed at the area of voice?**				
**Yes**		22	75.9	
**No**		7	24.1	
**Do you or did you have access to the bibliography on photobiomodulation in speech-language-hearing therapy or health in general?**				
**Yes**		26	89.7	
**No**		3	10.3	
**Do you or did you have access to the bibliography on laser therapy/photobiomodulation in the area of voice?**				
**Yes**		17	58.6	
**No**		12	41.4	
**Did you have access to CFFa recommendations on photobiomodulation use by speech-language-hearing therapists?**				
**Yes**		27	93.1	
**No**		2	6.9	
**What are the main cases in which you use laser therapy/photobiomodulation in voice?**				
**Behavioral dysphonia without lesions**	15			51.7
**Behavioral dysphonia with lesions**	13			44.8
**Organic neurological dysphonia**	9			31
**Dysphonia due to sequelae of head and neck cancer**	8			27.6
**Vocal improvement/training (speech)**	19			65.5
**Vocal improvement/training (singing)**	21			72.4
**Do you agree that speech-language-hearing therapists specialized in voice can use photobiomodulation?**				
**Yes**		27	93.1	
**No**		2	6.9	
**Do you agree that photobiomodulation is a therapeutic resource indicated for the voice?**				
**Yes**		29	100.0	
**No**		0	0.0	
**Do you use photobiomodulation in individuals with vocal fold inflammatory processes?**				
**Yes**		23	79.3	
**No**		6	20.7	
**Do you use photobiomodulation to improve/train vocal performance in speech?**				
**Yes**		24	82.8	
**No**		5	17.2	
**Do you use photobiomodulation to improve/train vocal performance in singing?**				
**Yes**		25	86.2	
**No**		4	13.8	
**What wavelength do you use the most in the laryngeal region?**				
**650 - 660 nm**		4	13.8	
**808 - 830 nm**		11	37.9	
**904 - 907 nm**		3	10.3	
**660 and 808 nm (simultaneously)**		11	37.9	
**What irradiation method do you use the most in the laryngeal region?**				
**Point contact**		29	100.0	
**Point non-contact**		0	0.0	
**Sweeping non-contact**		0	0.0	
**Where do you most often apply laser in the laryngeal region?**				
**Lamina of the thyroid cartilage unilaterally**		3	10.3	
**Lamina of the thyroid cartilage bilaterally**		23	79.4	
**Other**		3	10.3	
**How many points per laser application do you usually use for the voice?**				
**One**		0	0.0	
**Two**		11	37.9	
**Three**		4	13.8	
**Four**		6	20.7	
**Five or more**		8	27.6	
**Do you use systemic laser therapy - modified ILIB in the area of voice?**				
**Yes**		16	55.2	
**No**		13	44.8	
**Total**		29	100.0	

Only three (10.3%) of the participants learned about PBM in their undergraduate studies; most of them (22, 75.9%) learned about it in their postgraduate studies. Of the total sample, 28 (96.6%) took PBM training courses directed to voice clinical use; 26 (89.7%) had access to the bibliography on PBM use in SLH therapy or health; only 17 (58.6%) have/had access to the bibliography on PBM use in the voice; and 27 (93.1%) had access to CFFa recommendations on the topic.

All participants stated that PBM is a therapeutic resource indicated to the area of voice, and 27 (93.1%) of them said that SLH therapists specialized in voice can use it.

It was also found that all research participants use point contact irradiation and that 23 (79.4%) apply the laser on the lamina of the thyroid cartilage, bilaterally. Lastly, 16 (55.2%) participants stated using systemic laser therapy (ILIB) in the area of voice.

**Figure 1:**
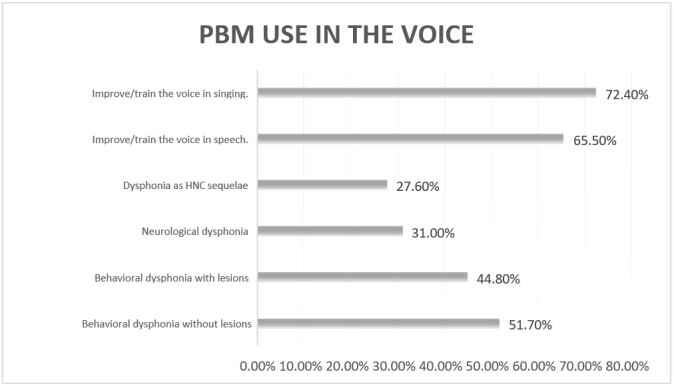
PBM use by participating speech-language-hearing therapists to treat the voice (n = 29)

As for purpose, 21 (72.4%) SLH therapists use PBM to improve/train singing voices; 23 (79.3%) use it in individuals with vocal fold inflammatory processes; 24 (82.8%) use it to improve vocal performance in speech; 25 (86.2%) use it to improve vocal performance in singing; 19 (65.5%) use it to improve/train the voice in speech; 15 (51.7%) use it in cases of behavioral dysphonia without lesions; 13 (44.8%) use it in cases of behavioral dysphonia with lesions; nine (31.0%) use it in cases of organic neurological dysphonia; and eight (27.6%) use it in cases of sequelae of head and neck cancer (Figure 1).

**Caption:** PBM = Photobiomodulation; HNC = Head and Neck Cancer

**Figure 2:**
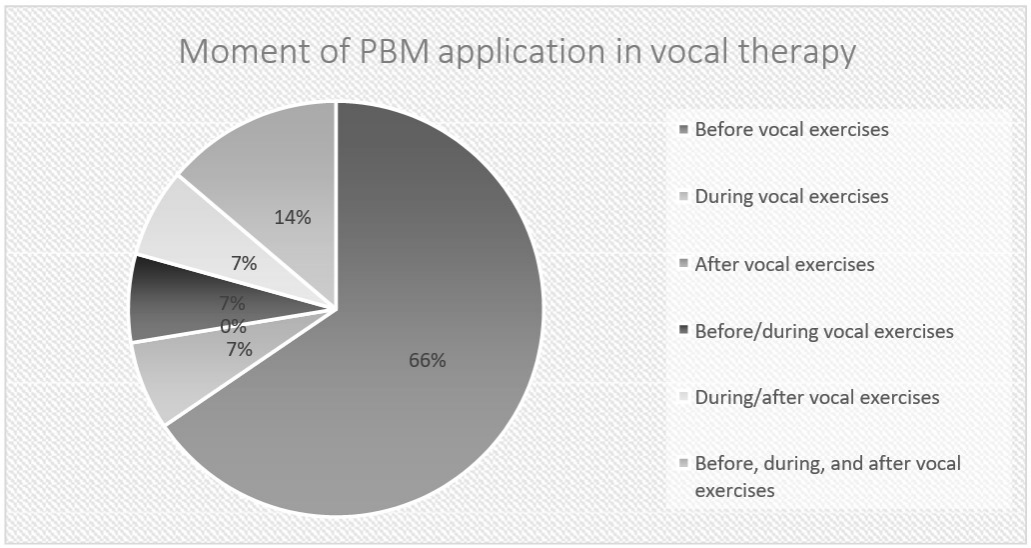
Moment of PBM application in vocal therapy by participating speech-language-hearing therapists (n = 29)

PBM is used by 19 (65.5%) participants before doing vocal exercises; 11 (37.9%) use 808-803 nm wavelengths; and another 11 (37.9%) use 660 and 808 nm simultaneously (Figure 2).

**Caption:** PBM = Photobiomodulation

**Figure 3:**
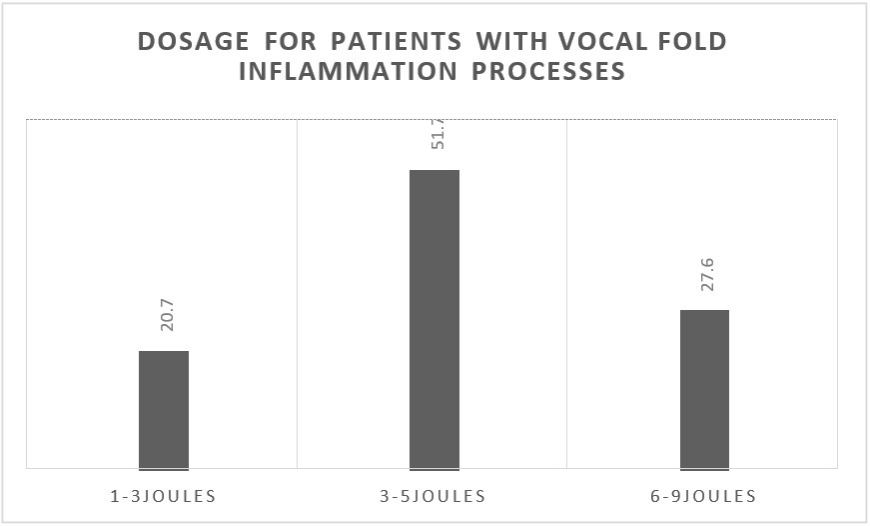
Dosage for patients with vocal fold inflammation processes (n = 29)

**Figure 4:**
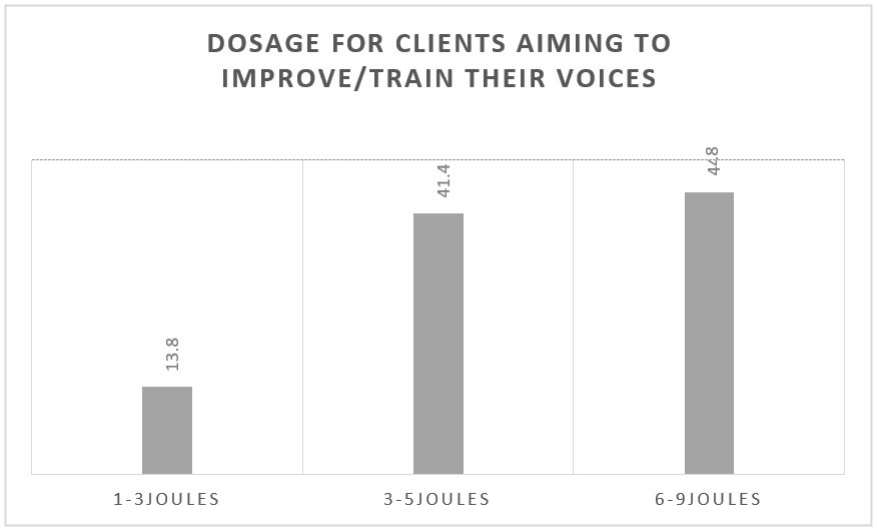
Dosage for clients aiming to improve/train their voices (n = 29)

Most participants use two points per laser application; 15 (51.7%) use 3-5 J doses for patients in vocal fold inflammatory processes (Figure 3), and 13 (44.8%) normally use 6-9 J doses to improve/train the voice (Figure 4).

## DISCUSSION

This study analyzed Brazilian SLH therapists’ knowledge and use of PBM in voice clinical practice. Most study participants were SLH therapists who specialized in voice but did not teach undergraduate or postgraduate courses. They reported knowing PBM principles and use in the area of voice. Moreover, most of them owned PBM equipment and normally used it in their clinical practice in the area of voice. This reinforces SLH therapists’ general interest in using new technology (such as PBM) in the therapeutic process, which involves learning the principles of these tools^(13)^.

PBM has been used as one of the strategies complementary to vocal therapy to either rehabilitate dysphonic individuals or improve/train occupational voice users. PBM use in dysphonic individuals is justified, as most phonotraumatic lesions have vocal fold edemas and inflammatory processes. As for occupational voice users with no laryngeal changes, PBM use is grounded on the possibility of optimizing the muscle energy mechanism associated with voice production, thus improving muscle performance, and decreasing the recovery time after using the voice in their occupation^(14,15)^. In both contexts, PBM is believed to modulate inflammation, maximize muscle performance, and, therefore, potentialize the effects of therapy or vocal training^(16)^.

PBM use in voice is incipient and grounded on translational premises from other areas and application in other body tissues. Hence, based on translational inference, PBM effects are expected to benefit vocal performance and modulate inflammatory processes with metabolic and photochemical actions in the mitochondria. Concerning the voice, improvements were verified in acoustic and aerodynamic measures and self-perceived vocal effort after using PBM^(5,12)^. However, the findings in these studies^(5,12)^ may not reflect the expected action mechanisms and effects, hypothesized by inference based on findings in other body tissues.

Most SLH therapists learned about PBM in postgraduate courses and/or extension training courses. It must be considered that PBM has been incorporated into SLH therapists’ clinical practice in the last years and that content on using technological resources complementary to conventional therapy is not necessarily among the primary objectives of the initial training of generalist professionals in their undergraduate studies^(13)^. Thus, such content is approached in extension and postgraduate specialization courses, which are part of SLH therapists’ continuing education.

CFFa states that SLH therapists can only use PBM as a therapeutic resource when specifically and adequately trained for it and are subject to legal responsibility in cases of malpractice, negligence, or recklessness. Adequate training generally has a minimum course load, obligatory content, practical training, and supervision to reinforce skills and competencies related to applying the technology^(17)^.

Clinical thinking is a continuous process of making decisions throughout treatment^(17)^. It includes a systematic and personalized approach to gathering information, formulating hypotheses, and selecting strategies. It is a complex process that requires professionals to have a well-established mindset. Thus, teaching tools must introduce students not only to knowledge but also training with scripts that make decision-making easier.

Therefore, PBM training in the area of voice involves not only general knowledge about PBM but also practical training and specific previous experience in the specialty. Clinical thinking to apply PBM to patients in voice clinical practice must involve deep knowledge of biological, etiological, and physiopathological aspects, and the main manifestations related to the condition being treated. Hence, training must include such knowledge, exposure to clinical cases, and practical training, preferably supervised. Training and supervision help consolidate knowledge for decision-making in each new clinical case^(17)^. It is recommended that initial basic training to use PBM be theoretical-practical, with a minimum 20-hour course load, also suggesting continuing education and in-depth training to use the resource. They also recommend minimum competencies SLH therapists must have by the end of their training.

Participants in this research reported having access to the bibliography on PBM use, though limited to the specific material in the area of voice. Specific literature in the area applied to cases of dysphonia and occupational voice use is still scarce^(5)^, with studies^(12,18)^ whose designs do not allow generalization and direct inferences for this field of practice.

Many participating SLH therapists had access to the CFFa recommendations on using low-level lasers in their profession. CFFa regulated PBM use as a therapeutic resource for SLH therapists in different areas. According to the resolution, they can use PBM therapy as a therapeutic resource associated with conventional SLH clinical procedures, directly (when applied to the specific region or location for its biostimulation) and/or indirectly (by applying ILIB, adapted or transdermic, for systemic intervention^(13)^. Due to the few studies on the effectiveness of PBM to treat dysphonic individuals, regulating it is necessary to legitimate and guide its exploratory use with a minimal assurance of not harming patients.

Most SLH therapists use PBM in the area of voice to improve/train it in speech or singing and to treat patients in vocal fold inflammatory processes. Using it in occupational voice users without vocal changes may be justified by the PBM effect of modulating metabolic processes in the mitochondria, leading to greater resistance to fatigue and less time to recover after intensely using the voice^(10,12)^.

The principle for using PBM in patients with vocal fold inflammatory lesions may be associated with the PBM effect on cell enzymes, changing the redox state, and increasing the oxidative chain mechanism in the mitochondria. Thus, it is expected to increase microcirculation, improve lymphatic drainage, increase epithelial cell proliferation and mobility, accelerate collagen synthesis, reduce the inflammatory response, and effectively heal tissues. All these factors would contribute to faster and more efficient vocal recovery^(12)^.

A smaller percentage of SLH therapists reported using PBM in neurological dysphonia (31%, n = 13) and dysphonia as a sequela of head and neck cancer (27.6%, n = 8). Concerning specifically the latter, though reported by fewer respondents, it is important to point out the relationship between risk, safety, and benefit to the patient in the clinical procedure.

Red and infrared PBM has proved to be safe and effective to manage the side effects of adjuvant cancer treatment^(8)^. In these cases, PBM can benefit the management of toxicities related to cancer treatment. On the other hand, there is not enough evidence available on PBM’s effect on malignant cell protection or increased tumoral growth^(8)^. Thus, professionals must communicate with patients about the potential PBM risks and benefits^(19)^.

In the area of voice, particularly regarding oncological contexts, PBM can be used to treat sequelae of head and neck cancer by managing symptoms in terms of reducing mucositis, xerostomia, lymphoedema, and trismus, and improving extrinsic laryngeal, tongue, and soft palate muscle performance^(20)^. In general, SLH therapists are recommended to judiciously evaluate PBM use to rehabilitate patients with sequelae of head and neck cancer. Furthermore, irradiation should be avoided in areas with neoplastic processes, as the literature available has no evidence of PBM effects on tumoral proliferation.

Participants reported that PBM is a therapeutic resource that can be indicated for use in the area of voice and that SLH therapists specialized in voice can use this practice. Despite the limited external evidence for the area, clinicians’ experience and patients’ preference for using such devices and the advances and discussions in the area justify the growing use of this technological resource complementary to traditional therapy^(13)^.

Concerning parameters to apply PBM in the area of voice, all SLH therapists use point contact irradiation in the laryngeal region. This seems to be the most adequate irradiation technique for this region, as it ensures greater light penetrability, greater precision of the irradiated energy, and low light reflection^(21)^. Moreover, the larynx has anatomical and histological specificities, such as the barriers (extrinsic muscles, skin and fat barrier, hyaline tissue of the thyroid cartilage) for the light to reach the laryngeal intrinsic muscles and the various vocal fold layers. Thus, it is hypothesized that point contact is the most adequate technique for this region^(20,22)^.

Participating SLH therapists reported applying PBM on the lamina of the thyroid cartilage bilaterally. The vocal folds are located inside the thyroid cartilage, and one of their insertion points is on this cartilage. Hence, applying it to the thyroid cartilage aims to reach the trilaminar structure of the vocal folds^(12)^. Some studies that used electrostimulation on the voice applied it on the lamina of the thyroid cartilage bilaterally, as it is appropriate for being nearer the vocal folds and the recurrent nerve, ensuring the stimulation of the laryngeal intrinsic muscles and vocal ligament, providing effective stimulation^(23)^. Besides this application point reported by most SLH therapists in this research, PBM can also address the voice by applying it to the submandibular region, thus irradiating the suprahyoid extrinsic muscles, and the intraoral region, specifically the soft palate^(5,24)^.

Most SLH therapists use PBM before doing vocal exercises. This use is probably justified by the need for precise irradiation on the adequate point and location. In general, vocal exercises that activate the glottal source or displace the articulators also move the laryngeal structure and may displace the predefined anatomical point (in this case, the vocal folds) and limit the expected effects^(16)^.

It is also indicated that irradiation be made 5 minutes to 6 hours before the activity when aiming at an acute effect, focusing on a single event^(25)^. On the other hand, when the goal is related to training strength and obtaining long-term (chronic) effects, irradiation must be made immediately before each exercise session^(25)^. When the goal is a chronic effect associated with resistance training, irradiation must occur mediately before and immediately after each exercise session^(25)^. In cases whose goal is tissue inflammation modulation, irradiation must also occur before exercises that recruit or manipulate the tissue inflammation area^(9,26)^. Thus, despite the lack of specific studies on the moment of irradiation to rehabilitate dysphonic patients or train occupational voice users, the evidence available leads to infer the indication of PBM use before the functional activity (vocal performance, for instance) or vocal exercise.

The 808-830 nm wavelength was the most reported by responding SLH therapists to apply in the area of voice. This wavelength corresponds to infrared light, which has a greater interaction with deeper tissue layers. Hence, infrared light is seemingly more adequate to overcome anatomical and histological barriers and reach the vocal folds^(2,11)^.

The dose of 3-5 J per point was the most reported by the respondents to apply in patients with vocal fold inflammation processes. The concept of PBM dose is directly related to the power of the equipment and the energy used at a point during the therapy session^(26,27)^. The energy corresponds to the equipment’s power multiplied by the irradiation time, resulting in a value in Joules. Obviously, calculating the dose of irradiated light that effectively reaches the tissue, involves other parameters, so the irradiated energy has been commonly used to describe doses in SLH clinical practice^(28)^. Even though 3-5 J per point was the most reported, there was a dispersion of the participants’ answers. This reinforces that PBM dosimetry is an important aspect to be discussed in the area, given the possibility of overdosage or underdosage, for example.

The indicated dose in oral-motor control is 3-4 J to provide analgesia in therapy and modulate inflammation in cases of temporomandibular disorder^(22,26)^. This dose is indicated to modulate the potential of the mitochondrial membrane of neurons, decreasing ATP generation, blocking sensory innervation, stimulating mitochondrial homeostasis, accelerating tissue healing, easing pain, and decreasing edema.

Most SLH therapists reported using 6-9 J to improve/train voices. There is a general tendency to use higher doses to improve muscle performance^(25,29)^. Higher doses stimulate bioenergetic pathways of the muscle fiber and the modulation of enzymes and oxygen-reactive species, which produces larger and more functional mitochondria, increasing oxygen consumption, and reducing muscle fatigue^(25,30)^.

SLH therapists reported irradiating two points in the laryngeal region in cases of dysphonia and vocal improvement. The number of points depends on variables such as muscle length, the place of irradiation, the amount of fat tissue, and so on^(25)^. Hence, given the neck dimensions and the said variables, consensus and future studies may verify the need for irradiation at more points in vocal clinical practice. It is essential to apply it on the whole extension of the target region or muscles to achieve biomodulation.

More than half of the SLH therapists (55.2%, n = 16) reported using ILIB in the area of voice. It consists of transdermic intravascular irradiation of a light beam in the radial artery. Such irradiation aims at the bloodstream to stimulate action in the whole organism. ILIB has potential generalized analgesic, spasmolytic, and sedative effects in almost all systems. However, there are yet no clinical studies supporting its use in patients with voice complaints or who seek vocal improvement.

PBM has been generally used in association with conventional vocal therapy to either rehabilitate dysphonic individuals or train vocal conditioning in speech or singing among occupational voice users. Data in this study portray this tool’s current use in vocal clinical practice and raise hypotheses of the possible justifications for its use in this context. Given the lack of robust external evidence, the specialists’ opinions may be a first step to understanding how a new tool is used.

Furthermore, the survey present in this manuscript may be a reference for experimental research and randomized clinical trials to verify the hypotheses related to PBM effects on dysphonic and vocally healthy individuals.

This exploratory study contributed to a cross-sectional understanding of specialists’ perception and use of PBM in their practice. It also has historical usefulness for future comparisons. The convenience sample may be representative of the reality investigated in a cross-section. A possible limitation of this study is the number of participants. Data were collected in 2020, the most critical part of the COVID-19 pandemic, which may have influenced the little adherence of participants. Moreover, future inquiries to this population may include questions on training duration and mandatory content to use PBM in vocal clinical practice.

## CONCLUSION

All participating SLH therapists reported knowing PBM precepts and use in the area of voice. Most respondents learned about PBM in their postgraduate studies. SLH therapists in the area of voice reported generally using point contact irradiation in the laryngeal region, applying PBM before vocal exercises, and using 808-830 nm wavelengths. Respondents used 3-5 J doses in patients with vocal fold inflammatory processes and 6-9 J doses in clients that aimed to improve/train their voices.
