# Supplementary material for: Opinion of speech-language pathologist on the use of photobiomodulation in the vocal clinic
Source: Codas. 2023 Sep 18;35(6):e20220060. doi: 10.1590/2317-1782/20232022060en (PMC10547142; doi:10.1590/2317-1782/20232022060en)
Supplement: Translation

# Translation: Opinião de fonoaudiólogos sobre uso da fotobiomodulação na clínica vocal

**DOI:** 10.1590/2317-1782/20232022060pt

**Authors:** Emerson Soares Pontes, Thays Garcia Vaiano, Roberto Sávio de Assunção Bastos, Leonardo Wanderley Lopes

**Affiliations:** 1 Universidade Federal da Paraíba - UFPB - João Pessoa (PB), Brasil.; 2 Centro de Estudos da Voz - CEV - São Paulo (SP), Brasil.; 3 Hospital Pronto Socorro Municipal Mário Pinotto - Belém (PA), Brasil.

**Keywords:** Voz, Terapia com Luz de Baixa Intensidade, Distúrbios da Voz, Treinamento da Voz, Doenças da Laringe

## Abstract

**Objetivo:**

investigar a opinião de fonoaudiólogos brasileiros sobre a formação, atuação e parâmetros utilizados para aplicação da fotobiomodulação (PBM) na clínica vocal.

**Método:**

utilizou-se uma *websurvey* no *Google Forms* composta por questões relacionadas à formação, atuação profissional e conhecimentos sobre a PBM na área de voz. Participaram 29 fonoaudiólogos, de ambos os sexos. Os dados foram analisados utilizando a estatística descritiva.

**Resultados:**

todos os participantes conheciam os fundamentos teóricos da PBM e entre eles, vinte e oito (96,6%) conheciam sua utilização especificamente na área de voz; vinte e cinco respondentes (86,2%) possuíam aparelho para fazer a irradiação e todos o utilizavam rotineiramente em sua prática clínica em voz. A maioria (28, 96,6%) participou de curso de capacitação em PBM, incluindo abordagens específicas para a área de voz. Os participantes afirmaram que a PBM é um recurso que pode ser utilizado na área de voz para melhorar a performance na voz cantada (25, 86,2%) e falada (24, 82,8%), além da sua aplicação em casos de processos inflamatórios nas pregas vocais (23, 79,3%). Quanto aos parâmetros de dosimetria, o comprimento de onda mais utilizado foi de 808 - 830nm (11, 37,9%) e 660/808nm simultaneamente (11, 37,9%), com dose de 3-5 J por ponto para os pacientes com processos inflamatórios nas pregas vocais (15, 51,7%,) e de 6-9 J (13, 44,8%) por ponto para os pacientes cujo objetivo era o aperfeiçoamento/condicionamento.

**Conclusão:**

os participantes do estudo demonstraram ter conhecimento e formação em PBM e suas aplicabilidades para a área de voz.

## INTRODUÇÃO

De modo geral, o objetivo da terapia vocal é reduzir os sintomas apresentados pelo paciente, reduzir sua limitação e melhorar a sua funcionalidade vocal para as situações cotidianas de uso da voz^(1-4)^. A terapia vocal envolve três componentes principais^(3,4)^: um alvo do tratamento, ou seja, a função do paciente que se deseja modificar pelo ingrediente; um ingrediente, que se refere à ação do clínico, uso de dispositivos, modelagem, uso de palavras e comandos, manipulações realizadas com o paciente durante o tratamento de reabilitação vocal, além dos elementos da metaterapia, todos voltados para modificar o alvo pré-definido; e o mecanismo de ação, ou seja, a compreensão sobre como o ingrediente irá modificar o alvo.

Os ingredientes da terapia vocal podem ser classificados em volitivos e não volitivos^(3,4)^. Em seu mecanismo de ação, os ingredientes volitivos incluem, necessariamente, a aprendizagem de novos comportamentos relacionados ao uso da voz. Por outro lado, ingredientes não volitivos não demandam uma ação específica do paciente (como executar um exercício, por exemplo) e a aprendizagem de um novo comportamento vocal (como modificar algum ajuste nos subsistemas respiratório, fonatório e ressonantal/articulatório, por exemplo).

Na terapia vocal, os ingredientes podem envolver a utilização de dispositivos tecnológicos como estratégia complementar modificar os alvos pré-definidos e alcançar os resultados terapêuticos esperados^(3,4)^. Nesse sentido, a fotobiomodulação (FBM), considerando a taxonomia proposta por Van Stan et al.^(4)^, pode ser considerado um dispositivo não volitivo (visto que o seu uso não demanda uma ação específica do paciente), utilizado para otimização dos resultados do tratamento^(5)^. A FBM consiste na capacidade da luz de induzir processos biológicos no nível celular, incluindo efeitos anti-inflamatórios, analgésicos, diminuição de edema, reparação tecidual e melhora no desempenho muscular^(6,7)^.

Na área de Voz, a FBM tem sido utilizada por suas propriedades anti-inflamatórias, analgésica e moduladora da atividade celular. Hipoteticamente, tais propriedades podem contribuir para diminuição do processo inflamatório, comumente presente nas lesões laríngeas, e melhorar o desempenho muscular dos pacientes. No entanto, até o momento, os efeitos são hipotéticos, por analogia aos efeitos sobre outros tecidos corporais não relacionados à laringe^(8-10)^. Ainda não existe corpo de evidência suficiente que suporte a recomendação da FBM em pacientes disfônicos ou profissionais da voz vocalmente saudáveis que desejem melhorar seu desempenho vocal. Nos últimos anos, observa-se um esforço para realização de pesquisas com o uso da FBM em voz^(11,12)^, embora ainda incipientes.

Por outro lado, a FBM tem se mostrado eficaz no tratamento de várias outras condições de saúde em áreas da odontologia, dermatologia, fisioterapia, otorrinolaringologia e fonoaudiologia^(11,13)^. Em 2021, o Conselho Federal de Fonoaudiologia (CFFa) normatizou o uso da FBM como recurso terapêutico a ser utilizado por fonoaudiólogos. De acordo com essa resolução, o fonoaudiólogo poderá utilizar a terapia por FBM como recurso terapêutico associado aos procedimentos clínicos fonoaudiológicos convencionais. Além disso, o tratamento poderá ser aplicado nas modalidades direta e/ou indireta, adaptada ou transdérmica para intervenção sistêmica^(13)^.

Sendo assim, considerando-se o possível potencial da FBM para otimizar os resultados da intervenção fonoaudiológica com pacientes disfônicos e com indivíduos vocalmente saudáveis, a escassez de estudos que suportem a evidência externa quanto ao uso da FBM na área de voz e a necessidade de compreender o uso corrente desse tipo de dispositivo na clínica vocal, o objetivo desta pesquisa foi investigar a opinião de fonoaudiólogos brasileiros sobre a formação, atuação e parâmetros utilizados para aplicação da fotobiomodulação (FBM) na clínica vocal. A partir dos resultados deste estudo, espera-se contribuir para o desenvolvimento de balizadores clínicos para uso da FBM na clínica vocal e fornecer *insights* para realização de pesquisas futuras.

## MÉTODO

### Desenho do estudo

Trata-se de um estudo observacional, transversal e quantitativo, aprovado pelo Comitê de Ética em Pesquisa com Seres Humanos do Centro de Ciências da Saúde da Instituição de origem, com o parecer de número 3.998.709. Todos os voluntários do estudo tiveram acesso e assinaram o Termo de Consentimento Livre e Esclarecido (TCLE), concordando em participar da pesquisa.

### Participantes

Para o recrutamento dos participantes, um *banner* com as informações e *link* de acesso para a pesquisa foi divulgado nas mídias sociais do Laboratório onde foi realizada esta investigação. Tais mídias possuem seguidores de diferentes regiões do país com foco de interesse específico na área de voz, o que favorece a captação da população-alvo da pesquisa.

Foram estabelecidos os seguintes critérios de elegibilidade: ser profissional graduado em Fonoaudiologia, atuante na área de Voz no Brasil. Dessa forma, foi constituída uma amostra por conveniência de 29 fonoaudiólogos, de ambos os sexos.

### Procedimentos

A pesquisa foi realizada por meio de uma *websurvey* hospedada na plataforma digital *Google Forms*. Inicialmente, foi realizada uma entrevista inicial com três fonoaudiólogos experientes na aplicação da FBM em Voz, discutindo os principais pontos que deveriam ser abordados para atingir os objetivos da presente pesquisa. Tais fonoaudiólogos cumpriram os seguintes critérios de elegibilidade: fonoaudiólogo especialista em voz com mais de 10 anos de atuação na área de Voz; ter participado de treinamento teórico-prático sobre o uso da FBM, incluindo suas aplicações na área de Voz; ter experiência mínima de dois anos na aplicação da FBM no processo de reabilitação de pacientes disfônicos ou no treinamento de profissionais da voz; ser especialista em Voz. Os três profissionais selecionados tinham atividade predominantemente clínica, sem vínculo de docência em universidades públicas ou privadas. A partir do conteúdo da entrevista foi elaborado o questionário que pode ser conferido no Quadro 1.

**Quadro 1:** Questões sobre fotobiomodulação e suas respectivas possibilidades de respostas

QUESTÃO	POSSIBILIDADE DE RESPOSTA DOS PARTICIPANTES
1. - Você conhece os preceitos da laserterapia/fotobiomodulação	Sim/Não
2. - Você conhece a utilização da laserterapia/fotobiomodulação na Fonoaudiologia, especificamente na área de voz?	Sim/Não
3. - Você tem acesso a um aparelho de Laser de baixa potência?	Não/Sim particular, sim de aluguel/empréstimo, sim aparelho da Universidade Outro
4. - Você costuma utilizar o laser na sua prática clínica na área de voz?	Sim/Não
5. - Você adquiriu conhecimentos da laserterapia/fotobiomodulação na graduação?	Sim/Não
6. - Você adquiriu conhecimentos da laserterapia/fotobiomodulação na Pós- graduação?	Sim/Não
7. - Você fez alguma formação/capacitação sobre laserterapia/fotobiomodulação em Fonoaudiologia ou em saúde?	Sim/Não
8. - Você fez alguma formação/capacitação sobre laserterapia/fotobiomodulação em Fonoaudiologia com direcionadores clínicos na área de Voz?	Sim/Não
9. - Você tem/teve acesso a alguma bibliografia relacionada a área de laserterapia/ Fotobiomodulação em Fonoaudiologia ou em saúde?	Sim/Não
10. - Você tem/teve acesso a alguma bibliografia relacionada a área de laserterapia/Fotobiomodulação na área de voz?	Sim/Não
11. - Você teve acesso às recomendações do CFFa sobre a utilização da laserterapia/ fotobiomodulação pelos fonoaudiólogos?	Sim/Não
12. - Quais os principais casos em que você faz mais o uso da laserterapia/ fotobiomodulação em voz?	Disfonia comportamental sem lesão / Disfonia comportamental com lesão / Disfonia por causas orgânicas neurológicas / Disfonia por sequelas de câncer de cabeça e pescoço / Aperfeiçoamento/Condicionamento vocal (Voz falada) / Aperfeiçoamento/Condicionamento vocal (Voz cantada).
13. - Você concorda que o fonoaudiólogo especialista em voz possa atuar em laserterapia/fotobiomodulação?	Sim/Não
14. - Você concorda que a laserterapia/fotobiomodulação é um recurso terapêutico indicado para a Voz?	Sim/Não
15. - Você utiliza a laserterapia/fotobiomodulação em indivíduos com processos inflamatórios nas pregas vocais (presença de edema, nódulos e/ou pólipo)?	Sim/Não
16. - Você utiliza a laserterapia/fotobiomodulação para melhorar a performance/ condicionamento vocal (voz falada)?	Sim/Não
17. - Você utiliza a Laserterapia/Fotobiomodulação para melhorar a performance/ condicionamento vocal (voz cantada)?	Sim/Não
18. - Em que momento da terapia vocal você utiliza a Laserterapia/ Fotobiomodulação?	Antes da execução de exercício vocal / Durante a execução de exercício vocal / Após a execução de exercício vocal / Antes, durante a execução de exercício vocal / Durante, após execução de exercício vocal / Antes, durante e após execução de exercício vocal
19. - Qual o comprimento de onda você mais utiliza na região de laringe?	650-660 nm / 808-830 nm / 904-907 nm / 660 e 808 nm (simultaneamente
20. - Qual o método de irradiação que você mais usa em região laríngea?	Contato pontual / Não-contato pontual / Não-contato varredura
21. - Qual local de aplicação do Laser/Led você mais utiliza na região laríngea?	Lâmina da cartilagem tireóidea unilateralmente / Lâmina da cartilagem tireóidea bilateralmente / outro
22. - Qual a quantidade de pontos por aplicação do Laser/Led você geralmente utiliza para a voz?	1 / 2 / 3 / 4 / Acima de 5
23. - Qual a dose em Joules (J) você utiliza para pacientes com processos inflamatórios nas pregas vocais?	1-3 J / 3-5 J / 6-9 J
24. - Qual a dose em Joules (J) você utiliza para pacientes de aperfeiçoamento/ condicionamento vocal?	1-3 J / 3-5 J / 6-9 J
25. - Você utiliza a Laserterapia sistêmica - ILIB Modificado/Transdérmico para a área de voz? no que concerne à reabilitação e/ou aperfeiçoamento/condicionamento vocal?	Sim/Não

Na sequência, o *link* de acesso ao questionário foi divulgado nas redes sociais do Laboratório onde foi realizada esta pesquisa, conforme descrito anteriormente. A coleta de dados foi realizada no período entre julho e agosto de 2020. O questionário continha itens sobre o perfil sociodemográfico dos fonoaudiólogos, dados de formação, atuação profissional e sobre os conhecimentos relacionados aos princípios e uso da FBM na área de voz. Antes de responder às questões, os participantes tiveram acesso ao texto explicativo sobre a pesquisa, procederam com a leitura do TCLE e, caso estivessem de acordo, prosseguiam com as respostas.

Os dados foram organizados e categorizados em planilhas do Microsoft Excel. Para análise dos dados, foi realizada análise estatística descritiva.

## RESULTADOS

Participaram da *websurvey* 29 fonoaudiólogos, cujas informações podem ser conferidas na Tabela 1.

**Tabela 1:** Dados sociodemográficos e profissionais dos fonoaudiólogos participantes da pesquisa

Variável	N	%
SEXO		
Feminino	22	75,9
Masculino	7	24,1
FAIXA ETÁRIA		
20 - 30 anos	9	31,0
31 - 40 anos	8	27,6
41 - 50 anos	8	27,6
51 - 60 anos	4	13,8
ESCOLARIDADE		
Graduação	3	10,3
Especialização	16	55,2
Mestrado	6	20,7
Doutorado	3	10,3
Sem resposta	1	3,4
ENSINO EM FONO		
Não Leciona	19	65,5
Leciona em Graduação	1	3,4
Leciona em Pós-Graduação	6	20,7
Ambos	1	3,4
Sem resposta	2	6,9
ENSINO EM PÓS-GRADUAÇÃO		
Não Leciona	19	65,5
Lato Sensu	5	17,2
Stricto Sensu	0	0,0
Ambos	1	3,4
Sem resposta	4	13,8
Total	29	100,0

Todos os participantes da pesquisa conheciam os fundamentos teóricos da FBM. Entre eles, 28 (96,6%) conheciam sua utilização especificamente na área de voz; 25 (86,2%) possuíam aparelho particular e todos costumavam utilizá-lo em sua prática clínica em voz (Tabela 2).

**Tabela 2:** Dados do questionário relacionado ao conhecimento sobre o uso da fotobiomodulação na área de voz

**Variável**		**N**	**%**	
**Você conhece os preceitos da Fotobiomodulação?**				
**Sim**		29	100,0	
**Não**		0	0,0	
**Você conhece a utilização da Fotobiomodulação especificamente na área de voz?**				
**Sim**		28	96,6	
**Não**		1	3,4	
**Você tem acesso a um aparelho de laser ou led?**				
**Não tenho acesso**		3	10,3	
**Sim, tenho um aparelho particular.**		25	86,2	
**Sim, de aluguel/empréstimo.**		0	0,0	
**Sim, tem um aparelho na Universidade/laboratório**		1	3,4	
**Você costuma utilizar o laser em sua prática clínica na área de voz?**				
**Sim**		25	86,2	
**Não**		4	13,8	
**Você adquiriu conhecimentos da Fotobiomodulação na graduação?**				
**Sim**		3	10,3	
**Não**		26	89,7	
**Você adquiriu conhecimentos da Fotobiomodulação na pós-graduação?**				
**Sim**		7	24,1	
**Não**		22	75,9	
**Você fez alguma capacitação em laserterapia?**				
**Sim**		28	96,6	
**Não**		1	3,4	
**Você fez alguma capacitação sobre Fotobiomodulação em fono com direcionadores clínicos para voz?**				
**Sim**		22	75,9	
**Não**		7	24,1	
**Você tem ou teve acesso a alguma bibliografia relacionada a área da Fotobiomodulação em fonoaudiologia ou em saúde?**				
**Sim**		26	89,7	
**Não**		3	10,3	
**Você tem/teve acesso a alguma bibliografia relacionada a área de Fotobiomodulação na voz?**				
**Sim**		17	58,6	
**Não**		12	41,4	
**Você teve acesso às recomendações do CFFa sobre a utilização da Fotobiomodulação pelos fonoaudiólogos?**				
**Sim**		27	93,1	
**Não**		2	6,9	
**Quais os principais casos em que você faz mais o uso da Laserterapia/Fotobiomodulação em voz?**				
**Disfonia comportamental sem lesão**	15			51,7
**Disfonia comportamental com lesão**	13			44,8
**Disfonia por causas orgânicas neurológicas**	9			31
**Disfonia por sequelas de Câncer de cabeça e pescoço**	8			27,6
**Aperfeiçoamento/condicionamento vocal (voz falada)**	19			65,5
**Aperfeiçoamento/condicionamento vocal (voz cantada)**	21			72,4
**Você concorda que o fonoaudiólogo especialista em voz possa atuar com a Fotobiomodulação?**				
**Sim**		27	93,1	
**Não**		2	6,9	
**Você concorda que a Fotobiomodulação é um recurso terapêutico indicado para voz?**				
**Sim**		29	100,0	
**Não**		0	0,0	
**Você utiliza a Fotobiomodulação em indivíduos com processos inflamatórios nas PPVV?**				
**Sim**		23	79,3	
**Não**		6	20,7	
**Você utiliza a Fotobiomodulação para melhorar a performance/condicionamento vocal (voz falada)?**				
**Sim**		24	82,8	
**Não**		5	17,2	
**Você utiliza a Fotobiomodulação para performance/condicionamento vocal (voz cantada)?**				
**Sim**		25	86,2	
**Não**		4	13,8	
**Qual o comprimento de onda você mais utiliza em região laríngea?**				
**650 - 660nm**		4	13,8	
**808 - 830nm**		11	37,9	
**904 - 907nm**		3	10,3	
**660 e 808nm (simultaneamente)**		11	37,9	
**Qual o método de irradiação você mais usa em região laríngea?**				
**Contato pontual**		29	100,0	
**Não-contato pontual**		0	0,0	
**Não-contato varredura**		0	0,0	
**Qual local de aplicação do laser você mais utiliza na região laríngea?**				
**Lâmina da cartilagem tireóidea unilateralmente**		3	10,3	
**Lâmina da cartilagem tireóidea bilateralmente**		23	79,4	
**Outro**		3	10,3	
**Qual a quantidade de pontos por aplicação do laser você geralmente utiliza para a voz?**				
**Um**		0	0,0	
**Dois**		11	37,9	
**Três**		4	13,8	
**Quatro**		6	20,7	
**Cinco ou mais**		8	27,6	
**Você utiliza a laserterapia sistêmica (ILIB) modificada para a área de voz?**				
**Sim**		16	55,2	
**Não**		13	44,8	
**Total**		29	100,0	

De todos os participantes, apenas três (10,3%) adquiriram conhecimentos sobre FBM na graduação; a maioria obteve tal conhecimento na pós-graduação (22, 75,9%). Do total da amostra, 28 (96,6%) fizeram curso de capacitação em FBM com direcionadores clínicos para voz; 26 (89,7%) tiveram acesso a alguma bibliografia relacionada a área da FBM em fonoaudiologia ou em saúde; somente 17 (58,6%) têm/tiveram acesso a alguma bibliografia relacionada a área de FBM na voz; e 27 (93,1%) tiveram acesso às recomendações do CFFa sobre o tema.

Todos os participantes afirmaram que a FBM é um recurso terapêutico indicado para a área de voz, e 27 (93,1%) deles referiram que o fonoaudiólogo especialista em voz pode atuar com esse recurso.

Foi observado também que todos os participantes da pesquisa utilizam o método de irradiação pelo contato pontual e que 23 (79,4%) fazem a aplicação do laser na lâmina da cartilagem tireóidea, bilateralmente. Por fim, 16 (55,2%) participantes afirmaram utilizar a laserterapia sistêmica (ILIB) para a área da voz.

Em relação à utilização da FBM pelos fonoaudiólogos, observou-se que 21 (72,4%) para aperfeiçoamento/condicionamento de voz (cantada); 23 (79,3%) utilizam em indivíduos com processos inflamatórios nas PPVV; 24 (82,8%) utilizam para melhorar a performance vocal (voz falada) e 25 (86,2%) para melhorar a performance vocal (voz cantada); 19 (65,5%) para aperfeiçoamento/condicionamento de voz (falada); 15 (51,7%) a utilizam em casos de disfonia comportamental sem lesão; 13 (44,8%) utilizam em casos de disfonia comportamental com lesão; nove (31,0%) em casos de disfonia por causas orgânicas neurológicas; oito (27,6%) em casos de sequela de CCP; (Figura 1).

**Figura 1:** Utilização da FBM na voz pelos fonoaudiólogos participantes (n = 29)

**Legenda:** FBM = Fotobiomodulação; CCP = Câncer de Cabeça e Pescoço

Do total de participantes, 19 (65,5%) fazem uso da FBM antes da execução do exercício vocal; 11 (37,9%) utilizam comprimentos de onda 808-803nm e outros 11 (37,9%) utilizam 660 e 808nm simultaneamente (Figura 2).

**Figura 2:** Momento de aplicação da FBM na terapia vocal pelos fonoaudiólogos participantes (n = 29)

**Legenda:** FBM = Fotobiomodulação

A maioria dos participantes utiliza dois pontos por aplicação do laser; 15 (51,7%) deles utilizam a dose de 3-5 J para pacientes com processos inflamatórios nas pregas vocais (Figura 3), e 13 (44,8%) costumam utilizar a dose de 6-9 J para aperfeiçoamento/condicionamento vocal (Figura 4).

**Figura 3:** Dosagem para pacientes com processos inflamatórios nas pregas vocais (n = 29)

**Figura 4:** Dosagem para clientes com objetivo de aperfeiçoamento/condicionamento vocal (n = 29)

## DISCUSSÃO

Este estudo analisou o conhecimento e o uso da FBM na clínica vocal por fonoaudiólogos brasileiros. A maioria dos participantes do estudo foi composta por fonoaudiólogos especialistas em voz e que não lecionam em cursos de graduação e de pós-graduação. Os participantes responderam que conhecem os princípios da FBM e da sua utilização na área de voz. Além disso, a maioria possui um aparelho de FBM e costuma utilizá-lo em sua prática clínica na área de voz. Tal fato reforça o interesse do fonoaudiólogo, de maneira geral, pelo uso de novas tecnologias como a FBM no processo terapêutico, o que envolve a busca pela compreensão dos princípios de tais ferramentas^(13)^.

A FBM tem sido utilizada como uma das estratégias complementares à terapia vocal, seja para reabilitação de indivíduos disfônicos ou para treinamento/condicionamento de profissionais da voz. A justificativa para o uso da FBM em indivíduos disfônicos é de que a maioria das lesões fonotraumáticas envolvem a presença de edema e processos inflamatórios nas pregas vocais. No contexto de profissionais da voz sem alteração laríngea, a aplicação da FBM está fundamentada na possibilidade de otimizar o mecanismo energético muscular associado à produção vocal, melhorando o desempenho muscular e reduzindo o tempo de recuperação após o uso profissional da voz^(14,15)^. Nesses dois contextos, a FBM atuaria no sentido de modular a inflamação, maximizar o desempenho muscular e, assim, potencializar o efeito da terapia ou treinamento vocal^(16)^.

O uso da FBM em voz é incipiente e sustentado por premissas translacionais de outras áreas e com aplicações em outros tecidos corporais. Nesse sentido, por inferência translacional, espera-se que os efeitos da FBM podem beneficiar o desempenho vocal e modular os processos inflamatórios com ações metabólicas e fotoquímicas em nível mitocondrial. Na área de voz, observou-se melhora nas medidas acústicas, aerodinâmicas e na autopercepção do esforço vocal após o uso da FBM^(5,12)^. No entanto, deve-se ressaltar que os achados dos estudos citados^(5,12)^ podem não traduzir, de fato, os mecanismos de ação e efeitos esperados que são hipotetizados por ilação a achados em outros tecidos corporais.

A maioria dos fonoaudiólogos adquiriu conhecimentos em FBM em cursos de pós-graduação e/ou em cursos livres de capacitação. Deve-se considerar que a FBM tem sido incorporada na prática clínica de fonoaudiólogos nos últimos anos e que, os conteúdos voltados ao uso de recursos tecnológicos complementares à terapia convencional, não estão, necessariamente, entre os objetivos primários da formação inicial de um profissional generalista ao término da graduação^(13)^. Dessa forma, tais conteúdos passam a ser abordados em cursos livres e em cursos de pós-graduação lato sensu, fazendo parte da educação continuada do fonoaudiólogo.

O CFFa preconiza que o fonoaudiólogo só poderá utilizar o recurso terapêutico de FBM quando possuir capacitação específica e adequada, estando sujeito à responsabilidade legal em casos de imperícia, negligência e imprudência. De maneira geral, uma capacitação adequada deve envolver carga horária mínima, conteúdos obrigatórios, assim como treinamento e supervisão para fortalecer as habilidades e competências relacionadas à aplicação de uma tecnologia^(17)^.

O raciocínio clínico é um processo contínuo de tomada de decisão ao longo de um atendimento^(17)^. Ele inclui uma abordagem sistemática e personalizada de coleta de informações, formulação de hipóteses e seleção de estratégias. Ele é um processo complexo e exige que os esquemas mentais estejam sedimentados no profissional. Dessa forma, as ferramentas de ensino devem possibilitar não somente a exposição do aprendiz ao conhecimento, mas o treinamento em scripts que facilitem a tomada de decisão.

Nesse sentido, a capacitação em FBM para a área de voz envolve não somente o conhecimento geral sobre FBM, mas a formação e experiência prévia específica da especialidade. O raciocínio clínico para a aplicação da FBM em pacientes na clínica vocal deve envolver um profundo conhecimento dos aspectos biológicos, etiológicos, fisiopatológicos e das principais manifestações envolvidas com a condição tratada. Sendo assim, a capacitação deveria envolver tal conhecimento, a exposição aos casos clínicos e o treinamento prático, preferencialmente, sob supervisão. O treinamento e a supervisão são facilitadores para que esse conhecimento seja encapsulado para a tomada de decisão a cada novo caso clínico^(17)^. Recomenda-se que a formação básica inicial para uso da FBM seja teórico-prática, com carga-horária mínima de 20h, com sugestão de educação continuada e necessidade de aprofundamento quanto ao uso desse recurso. Além disso, recomenda competências mínimas que o fonoaudiólogo deverá apresentar ao final da formação.

Os participantes desta pesquisa referiram ter acesso à material bibliográfico relacionado ao uso da FBM, embora com acesso restrito a materiais específicos na área de voz. Ressalta-se que a literatura específica da área aplicada aos casos de disfonia e voz profissional ainda é escassa^(5)^, com estudos^(12,18)^ cujo delineamento não permite generalizações e inferências diretas para esses campos de atuação.

Dos fonoaudiólogos participantes, grande parte teve acesso às recomendações do CFFa sobre a utilização do laser de baixa potência na profissão. O CFFa normatizou o uso da FBM como recurso terapêutico a ser utilizado por fonoaudiólogos em diferentes áreas. De acordo com a resolução, o fonoaudiólogo poderá utilizar a terapia por FBM como recurso terapêutico associado aos procedimentos clínicos fonoaudiológicos convencionais, aplicado nas modalidades direta quando aplicado na região ou local específico para promover a bioestimulação e/ou indireta com a aplicação do ILIB, adaptada ou transdérmica para intervenção sistêmica^(13)^. Considerando-se a escassez de estudos sobre a efetividade da FBM no tratamento de indivíduos disfônicos, a regulamentação é necessária para legitimar e nortear o uso em caráter exploratório, com a mínima garantia de não maleficência ao paciente.

Quanto à utilização da FBM pelos fonoaudiólogos na área de voz, a maioria utiliza em indivíduos cujo objetivo é o aperfeiçoamento/condicionamento da voz falada e cantada, assim como em pacientes com processos inflamatórios nas pregas vocais. A justificativa para o uso em profissionais da voz sem alteração vocal pode estar associada ao efeito da FBM em modular os processos metabólicos em nível mitocondrial, refletindo em maior resistência à fadiga e menor tempo de recuperação após o uso intensivo^(10,12)^.

O princípio para utilização da FBM em pacientes com lesões inflamatórias nas pregas vocais pode estar associado ao efeito da FBM sobre as enzimas celulares e por alterar o estado redox, aumentando o mecanismo da cadeia oxidativa em nível mitocondrial. Dessa forma, espera-se que haja aumento na microcirculação, melhora a drenagem linfática, aumenta a proliferação e mobilidade das células epiteliais, aceleração da síntese de colágeno, redução da resposta inflamatória e a cicatrização do tecido de forma efetiva. Todos esses fatores contribuiriam para uma recuperação vocal mais eficiente e rápida^(12)^.

Um menor percentual de fonoaudiólogos referiu utilizar a FBM em disfonias de causa neurológica (31%, n = 13) e nas disfonias por sequela de câncer de cabeça e pescoço (27,6%, n = 8). Especificamente quanto ao último caso, embora referido por um número menor de respondentes, deve-se comentar acerca das relações entre risco, segurança e benefícios da ação clínica sobre o paciente.

A FBM (no espectro vermelho ou infravermelho) demonstrou ser segura e efetiva para o manejo dos efeitos colaterais relacionados ao tratamento adjuvante do câncer^(8)^. Nesses casos, a FBM pode ser uma abordagem que beneficia no manejo das toxicidades relacionadas ao tratamento do câncer. Por outro lado, ainda não há evidência disponível suficiente quanto ao efeito da FBM na proteção celular maligna ou no aumento do crescimento tumoral^(8)^. Nesse sentido, é mandatório que os profissionais comuniquem os pacientes sobre os potenciais riscos e benefícios relacionados à FBM^(19)^.

Na área de voz, especificamente no contexto oncológico, a FBM pode vir a ser utilizada no tratamento das sequelas de câncer de cabeça e pescoço para o manejo de sintomas relacionadas aos alvos terapêuticos de: redução da mucosite, redução da xerostomia, redução do linfedema, redução do trismo, melhora no desempenho da musculatura extrínseca da laringe, melhora no desempenho da musculatura da língua e do palato mole^(20)^. De maneira geral, recomenda-se que o fonoaudiólogo avalie criteriosamente o uso da FBM na reabilitação de pacientes com sequelas de câncer de cabeça e pescoço. Além disso, deve evitar a irradiação sobre áreas com processos neoplásicos, considerando-se que, na literatura disponível, não há evidência sobre os efeitos da FBM na proliferação tumoral.

Os participantes referiram que a FBM é um recurso terapêutico que pode ser indicado para utilização na área de voz e que os fonoaudiólogos especialistas em voz podem atuar com essa prática. Mesmo com evidências externas limitadas para a área, a experiência dos clínicos e a preferência dos pacientes pela utilização desses dispositivos, como também avanços e discussões na área, tem justificado o uso crescente desse recurso tecnológico complementar à terapia tradicional^(13)^.

Quando questionados sobre os balizadores de aplicação da FBM na área de voz, todos os fonoaudiólogos utilizam a técnica de irradiação por contato pontual em região laríngea. A técnica pontual por contato parece ser mais adequada para a irradiação na região laríngea, uma vez que permite uma maior penetrabilidade da luz, maior precisão da energia irradiada e baixa reflexão da luz^(21)^. Além disso, a laringe tem especificidades anatômicas e histológicas, tais como as barreiras (musculatura extrínseca, barreira dérmica e adiposa, tecido hialino da cartilagem tireóide) até que a luz alcance os músculos intrínsecos da laringe e as diferentes camadas das pregas vocais. Dessa forma, hipotetiza-se que a técnica pontual por contato seria mais adequada para utilização nessa região^(20,22)^.

Os fonoaudiólogos participantes referiram aplicar a FBM na lâmina da cartilagem tireóidea bilateralmente. As pregas vocais situam-se no interior da cartilagem tireóide, tendo um de seus pontos de inserção nessa cartilagem. Dessa forma, a aplicação na cartilagem tireóide tem por objetivo alcançar a estrutura trilaminar das pregas vocais^(12)^. Alguns estudos que utilizaram a eletroestimulação na voz aplicaram esta técnica nas lâminas da cartilagem tireóidea bilateralmente por ser uma localização oportuna, por ser mais próxima das pregas vocais e do nervo recorrente, garantindo uma estimulação na musculatura intrínseca da laringe, ligamento vocal propiciando uma estimulação efetiva^(23)^. Além desse ponto de aplicação referido pela maioria dos fonoaudiólogos nesta pesquisa, a FBM também pode ser aplicada em voz na região submandibular, para irradiação dos músculos extrínsecos supra-hioideos e na região intraoral, especificamente, no palato mole^(5,24)^.

A maioria dos fonoaudiólogos utilizam a FBM antes da execução dos exercícios vocais. A provável justificativa para esse uso pode estar relacionada à necessidade de precisão da irradiação, no ponto e local adequado. De maneira geral, a execução dos exercícios vocais com ativação da fonte glótica ou deslocamento dos articuladores movimenta o arcabouço laríngeo e pode deslocar o ponto anatômico pré-definido (nesse caso, as pregas vocais) e limitar o alcance dos efeitos esperados^(16)^.

Além disso, há indicação para que a irradiação seja realizada entre cinco minutos a seis horas antes da atividade, quando o objetivo é um efeito agudo, focado em um evento único^(25)^. Por outro lado, quando o objetivo está relacionado ao treinamento de força e ao efeito de longo prazo (crônico), a irradiação deve ser realizada imediatamente antes de cada sessão de exercício^(25)^. Nos casos que o objetivo é o efeito crônico associado ao treinamento de resistência, a indicação é que a irradiação deva ocorrer imediatamente antes e imediatamente após cada sessão de exercício^(25)^. Para os casos em que o objetivo é a modulação da inflamação tecidual, a irradiação também é recomendada antes da execução de um exercício que envolva o recrutamento ou manipulação da área com inflamação tecidual^(9,26)^. Sendo assim, embora não haja estudos específicos quanto ao momento da irradiação na reabilitação de disfônicos ou treinamento de vozes profissionais, por ilação, a evidência disponível suporta a indicação do uso da FBM antes da atividade funcional (performance vocal, por exemplo) ou do exercício vocal.

O comprimento de onda 808-830nm foi o mais referido pelos fonoaudiólogos respondentes para aplicação na área de voz. Tal comprimento corresponde à luz infravermelha, que possui maior interação com camadas de tecido mais profundas. Dessa forma, a luz infravermelha parece ser mais adequada para ultrapassar as barreiras anatômicas e histológicas para atingir o nível das pregas vocais^(2,11)^.

A dose de 3-5 J por ponto foi a mais referida pelos respondentes para a aplicação em pacientes com processos inflamatórios nas pregas vocais. O conceito de dose em FBM está diretamente relacionado à potência do equipamento utilizado e à energia que será utilizada em um ponto durante a sessão de terapia^(26,27)^. Dessa forma, a energia corresponde à multiplicação da potência do equipamento pelo tempo de irradiação, resultando em um valor dado em Joules. Obviamente, o cálculo da dose efetiva de luz irradiada que é entregue ao tecido envolva outros parâmetros, de modo que a energia irradiada tem sido utilizada comumente para a descrição da dose na clínica fonoaudiológica^(28)^. Embora 3-5 J por ponto tenha sido o valor mais referido, observou-se que há uma dispersão nas respostas dos participantes. Tal fato reforça que a dosimetria da FBM é um dos aspectos importantes a ser discutido na área, considerando a possibilidade de super ou subdosagem, por exemplo.

Na área de motricidade orofacial há uma indicação para o uso de doses de 3-4 J associado ao objetivo terapêutico de analgesia e modulação da inflamação em casos de disfunção temporomandibular^(22,26)^. Essa dose é preconizada para modular o potencial da membrana mitocondrial nos neurônios, levando à diminuição da geração de ATP, bloqueio da inervação sensorial, estímulo da homeostase mitocondrial, aceleração da cicatrização tecidual e redução da dor e edema.

A maioria dos fonoaudiólogos referiram utilizar a dosagem de 6-9 Joules para os casos de aperfeiçoamento/condicionamento vocal. De maneira geral, observa-se uma tendência ao uso de doses mais altas quando o objetivo está associado à melhora do desempenho muscular^(25,29)^. A dosagem mais alta estimula as vias bioenergéticas das fibras musculares, a modulação enzimática e a modulação de espécies reativas de oxigênio, o que produz mitocôndrias maiores e mais funcionais, aumentando o consumo de oxigênio e reduzindo a fadiga muscular^(25,30)^.

Os fonoaudiólogos referiram irradiar dois pontos na região laríngea nos casos de disfonia e aperfeiçoamento vocal. A quantidade de pontos depende de variáveis tais como, a extensão do músculo, o local de irradiação, a quantidade de tecido adiposo, entre outros^(25)^. Sendo assim, considerando as dimensões do pescoço e as variáveis citadas, consensos e estudos futuros podem verificar a necessidade de irradiação em mais pontos quando a aplicação for realizada na clínica vocal. A aplicação em toda a extensão da região alvo ou musculatura que se deseja alcançar é essencial para se obter um efeito biomodulador.

Mais da metade dos fonoaudiólogos (55,2%, n = 16) referiram utilizar o ILIB na área de voz, essa técnica consiste na irradiação intravascular do sangue, que é feita transdermicamente, por meio de um feixe luminoso na artéria radial. O objetivo é irradiar a corrente sanguínea e, com isso estimular a ação do organismo como um todo. O ILIB tem potenciais efeitos analgésicos, espasmolíticos e sedativos generalizados em quase todos os sistemas. No entanto, ainda não existem estudos clínicos que dêem suporte à utilização da técnica ILIB em pacientes com queixa de problema de voz ou para aperfeiçoamento vocal.

De maneira geral a FBM tem sido utilizada associada à terapia vocal convencional, seja na reabilitação de indivíduos disfônicos ou treinamento para condicionamento vocal de profissionais da voz falada ou cantada. As informações do presente estudo permitem ter uma visão acerca do uso atual dessa ferramenta na clínica vocal, assim como hipotetizar sobre as possíveis justificativas do seu uso nesse contexto. Na ausência de evidências externas robustas, a opinião dos especialistas pode ser um primeiro passo para compreender o uso de uma nova ferramenta.

Além disso, o levantamento apresentado neste manuscrito pode servir de base para a realização de pesquisas experimentais e ensaios clínicos randomizados para verificar as hipóteses relacionadas aos efeitos da FBM em indivíduos disfônicos e vocalmente saudáveis.

Esse estudo tem um caráter exploratório, contribuindo para a compreensão transversal sobre como os especialistas têm compreendido e utilizado a FBM em sua prática. Além disso, tem a sua utilidade histórica, por permitir comparações no futuro. Sendo assim, a amostra por conveniência recrutada pode ser representativa da realidade investigada em um recorte transversal. Uma das possíveis limitações do presente estudo pode ser o número de participantes. A coleta de dados aconteceu no ano de 2020, em meio à pandemia pela COVID-19 no seu momento mais crítico. Tal fato pode ter influenciado para a reduzida adesão dos participantes. Além disso, inquéritos futuros com essa população podem incluir entre as suas questões a duração e o conteúdo obrigatório na capacitação para o uso da FBM na clínica vocal.

## CONCLUSÃO

Todos os fonoaudiólogos participantes relataram conhecer os preceitos da FBM, bem como a sua utilização na área de Voz. A maioria dos respondentes adquiriu conhecimentos sobre a FBM em uma pós-graduação. Na área de Voz, os fonoaudiólogos referiram que, de maneira geral, utilizavam o método de irradiação por contato pontual em região laríngea, com aplicação da FBM antes da execução dos exercícios vocais e com o comprimento de onda 808-830nm. Os respondentes utilizavam uma dose de 3-5 Joules para os casos de pacientes com processos inflamatórios nas pregas vocais, e 6-9 Joules como parâmetros dosimétricos para clientes cujo objetivo é o aperfeiçoamento/condicionamento vocal.
